# Draft Genome Sequence of *Pseudomonas psychrophila* MTCC 12324, Isolated from the Arctic at 79°N

**DOI:** 10.1128/genomeA.00578-15

**Published:** 2015-06-11

**Authors:** Wilson Peter Abraham, Sabu Thomas

**Affiliations:** Cholera and Biofilm Research Lab, Rajiv Gandhi Centre for Biotechnology, Trivandrum, Kerala, India

## Abstract

*Pseudomonas psychrophila* MTCC 12324 is a facultatively psychrophilic bacterium isolated from the Arctic fjord Ny-alesund in the Svalbard Archipelago. Here, we present a 5.2-Mb draft genome sequence of *P. psychrophila* MTCC 12324, reported for the first time, from the Arctic. This enables a study of the cold adaptation mechanisms to survive under the extreme cold conditions.

## GENOME ANNOUNCEMENT

The polar Arctic region is an unexplored area of the earth, and that environment possesses microorganisms that permanently thrive at temperatures at and around the freezing point of water. In the present investigation, a Gram-negative rod-shaped bacterium, *Pseudomonas psychrophila* MTCC 12324 (also known as RGCB 166), was isolated from fjord water in the Arctic having a temperature of 2°C. The test organism was identified as *P. psychrophila* MTCC 12324 by sequencing its 16S rRNA gene (GenBank accession no. JX271040.1). The bacterium grows at a temperature range between 4°C and 30°C, with optimal growth at a temperature range of 20 to 25°C. Its growth pattern indicates that the test organism falls in the category of being facultatively psychrophilic. Previously, a cold-adapted sulfamethoxazole-degrading *P. psychrophila* strain was isolated from sewage effluent in China (1), and another isolate of *P. psychrophila* was reported from cold storage in Japan (2). In the present study, we report *P. psychrophila* MTCC 12324, isolated from the Arctic, for the first time.

The genome of *P. psychrophila* MTCC 12324 was sequenced using an Illumina HiSeq 2000 and an Illumina MiSeq system. The reads were assembled into 150 large contigs (>2,330 bp) using ABySS (3), SPAdes (4), Velvet (5), and SOAP*denovo*2 (6). The contig *N*_50_ is approximately 57.3 kb, and the largest contig assembled is approximately 235.3 kb. The draft sequence consists of 5,269,174 bases, with a mean G+C content of 57.52%. A total of 5,036 coding sequences were predicted using Glimmer (7), and 51 structural RNAs were identified from the contigs using tRNAscan-SE (8). Several genes pertaining to cold adaptation, such as fatty acid desaturase, polynucleotide phosphorylase, and peptidyl-prolyl *cis*-*trans* isomerase, have been identified from the sequenced genome of *P. psychrophila* MTCC 12324. Genomic information acquired through whole-genome sequencing will shed light on the complicated cold adaptation mechanisms of bacteria, which are very diverse in nature.

### Nucleotide sequence accession numbers.

This whole-genome shotgun project has been deposited in GenBank under the accession no. LBHT00000000. The version described in this paper is the first version, LBHT00000000.1

## References

[1] Jiang B, Cui D, Li A, Gai Z, Ma F, Yang J, Ren N 2012 Genome sequence of a cold-adaptable sulfamethoxazole-degrading bacterium, *Pseudomonas psychrophila* HA-4. J Bacteriol 194:5721. doi:10.1128/JB.01377-12. 23012293 PMC3458689

[2] Yumoto I, Kusano T, Shingyo T, Nodasaka Y, Matsuyama H, Okuyama H 2001 Assignment of *Pseudomonas* sp. strain E-3 to *Pseudomonas psychrophila* sp. nov., a new facultatively psychrophilic bacterium. Extremophiles 5:343–349. doi:10.1007/s007920100199. 11699648

[3] Simpson JT, Wong K, Jackman SD, Schein JE, Jones SJ, Birol I 2009 ABySS: a parallel assembler for short read sequence data. Genome Res 19:1117–1123. doi:10.1101/gr.089532.108. 19251739 PMC2694472

[4] Bankevich A, Nurk S, Antipov D, Gurevich AA, Dvorkin M, Kulikov AS, Pevzner PA, Nikolenko SI, Pham S, Prjibelski AD, Pyshkin AV, Sirotkin AV, Vyahhi N, Tesler G, Alekseyev MA, Pevzner PA 2012 SPAdes: a new genome assembly algorithm and its applications to single-cell sequencing. J Comput Biol 19:455–477. doi:10.1089/cmb.2012.0021. 22506599 PMC3342519

[5] Zerbino DR, Birney E 2008 Velvet: algorithms for *de novo* short read assembly using de Bruijn graphs. Genome Res 18:821–829. doi:10.1101/gr.074492.107. 18349386 PMC2336801

[6] Luo R, Liu B, Xie Y, Li Z, Huang W, Yuan J, Wang J, Chen Y, Pan Q, Liu Y, Tang J, Wu G, Zhang H, Shi Y, Liu Y, Yu C, Wang B, Lu Y, Han C, Cheung DW 2012 SOAP*denovo*2: an empirically improved memory-efficient short-read *de novo* assembler. GigaScience 1:18. doi:10.1186/2047-217X-1-18. 23587118 PMC3626529

[7] Delcher AL, Harmon D, Kasif S, White O, Salzberg SL 1999 Improved microbial gene identification with Glimmer. Nucleic Acids Res 27:4636–4641. doi:10.1093/nar/27.23.4636. 10556321 PMC148753

[8] Lowe TM, Eddy SR 1997 tRNAscan-SE: a program for improved detection of transfer RNA genes in genomic sequence. Nucleic Acids Res 25:955–964. doi:10.1093/nar/25.5.0955. 9023104 PMC146525

